# Selective recruitment of γδ T cells by a bispecific antibody for the treatment of acute myeloid leukemia

**DOI:** 10.1038/s41375-021-01122-7

**Published:** 2021-02-01

**Authors:** Rajkumar Ganesan, Vijaykumar Chennupati, Balaji Ramachandran, Michael Riis Hansen, Sanjaya Singh, Iqbal S. Grewal

**Affiliations:** 1 Janssen Biotherapeutics, The Janssen Pharmaceutical Companies of Johnson & Johnson, Ambler, PA USA; 2 Syngene International Ltd., Bangalore, India

**Keywords:** Targeted therapies, Cancer immunotherapy

## Abstract

Despite significant progress over the last few decades in the treatment of acute myeloid leukemia (AML), there still remains a major unmet medical need for this disease. Immunotherapy approaches for redirecting pan CD3^+^ T cells to target leukemia blasts have shown limited efficacy in clinical trials and often accompanied with severe toxicity in AML patients. We designed an alternative engager molecule (Anti-TRGV9/anti-CD123), a bispecific antibody that can simultaneously bind to the Vγ9 chain of the Vγ9Vδ2^+^ γδ T cell receptor and to AML target antigen, CD123, to selectively recruit Vγ9^+^ γδ T cells rather than pan T cells to target AML blasts. Our results suggest that prototypic bispecific antibodies (a) selectively activate Vγ9^+^ γδ T cells as judged by CD69 and CD25 surface expression, and intracellular Granzyme B expression, (b) selectively recruit Vγ9^+^ γδ T cells into cell–cell conjugate formation of γδ T cells with tumor cells indicating selective and effective engagement of effector and target tumor cells, and (c) mediate γδ T cell cytotoxicity (in vitro and in vivo) against tumor antigen-expressing cells. Collectively, these findings suggest that selectively redirecting Vγ9^+^ γδ T cells to target AML blasts has a potential for immunotherapy for AML patients and favors further exploration of this concept.

## Introduction

Acute myeloid leukemia (AML) is a fast-growing disease that occurs in large, immature white blood cells leading to leukemia [1]. Chemotherapy and hematopoietic stem cell transplantation are some treatments options; however, ineligible patients survive only for less than a year [2, 3]. As the role of T cells in the anti-cancer immune response is strongly supported and correlates with a favorable clinical prognosis in many cancers [4], novel strategies to redirect T cells for immunotherapy have been increasingly investigated for multiple cancers including AML [5–8]. Two different strategies, chimeric antigen receptor (CAR) T cells and bispecific protein therapeutics to recruit CD3^+^ T cells to kill tumor cells, are currently being explored in clinical trials. Although redirecting T cells via CD3 is attractive, it raises two key concerns: (i) indiscriminate stimulation of pan-T cells including various immunoregulatory and immunosuppressive T cells, which play an active role in immune evasion and (ii) overt pan T cell activation that can lead to cytokine storm resulting in severe side effects. To alleviate some of these limitations, an alternative strategy would be to selectively redirect only cytotoxic cells rather than indiscriminately stimulate pan-T cells.

Recently, γδ T cells have emerged as a great player in the cancer immunotherapy field [9, 10]. Although γδ T cells are ~5% of human peripheral blood T cells, they are the majority in epithelial tissues (lung, gut, and genital tract) [11] and display both innate and adaptive characteristics [12]. In addition, γδ T cells have been shown to infiltrate cancers and positively correlate with favorable clinical outcome [4]. In adults, 50 to >95% of blood γδ T cells usually express a TCR encoded by Vγ9 and Vδ2 chains, and are endowed with potent anti-cancer functions [8, 13]. Interestingly, anti-Vγ9/Vδ2 TCR antibodies that bind to Vγ9 or Vδ2 chain were shown to activate these γδ T cells [14, 15], suggesting that anti-TCR-based redirecting molecules can be constructed to redirect Vγ9/Vδ2^+^ TCR γδ T cell subset for tumor elimination. Non-Fc scaffold-based bispecific antibodies with tumor targets such as Her2/EGFR were described recently [9, 10, 15]. Thus, approaches that help to overcome the limitations of CD3-based redirection and avoid pan activation of T cells can be designed to induce potent tumor cell lysis by selectively recruiting γδ T cells.

In the present study, we designed and characterized a bispecific antibody, anti-TRGV9/anti-CD123, that can simultaneously bind to the Vγ9 chain of the γδ T cell receptor and to CD123. CD123 is a validated target antigen expressed abundantly on AML blasts, and on CD34^+^/CD38^-^ leukemic stem cells, and thus provides an opportunity to target malignant cells. Our results presented in this report suggest that selectively redirecting Vγ9^+^ γδ T cells to target AML blasts has a potential for immunotherapy for AML patients.

## Materials and methods

Blood collection, isolation of PBMCs, expansion of Vγ9^+^ γδ T cells, tumor cell lines and reagents, flow cytometer methods, statistical analysis, and engineering of the bispecific antibodies are described in the Supplementary information.

### Bispecific antibody binding assay

Binding of anti-TRGV9/CD123 (Vγ9/CD123) and anti-TRGV9/ Null (Vγ9/Null) bispecific antibodies to CD123-expressing cell lines and γδ T cells was carried out by flow cytometry. Briefly, cells were stained with bispecific antibodies and detected by labelled mouse anti-human IgG1 or IgG4 secondary antibody (SouthernBiotech, Birmingham, and AL), and the fluorescence of stained cells was measured on a flow cytometer.

### Cell–cell conjugate formation assay

Enriched effector cells (γδ T cells) and target cells (Kasumi-3) were labelled with 0.3 µM CellTracker^TM^ Green CMFDA and 1.5 µM CellTracker^TM^ Orange CMRA (Life Technologies, Carlsbad, CA) dyes, respectively. 1.0 × 10^5^  cells/mL labelled cells of each were co-cultured in the presence or absence of specified bispecifics at a concentration of 1 µg/mL and incubated at 37 °C, 5% CO_2_ for 1 h one hour. Cells were fixed by incubating them with BD Cytofix (BD Biosciences, San Jose, CA) for 15 min at 4 °C and analyzed on flow cytometer. FlowJo analysis software (Treestar Inc, Ashland, OR) was used to analyze cell–cell conjugate formation. Cells engaged in cell–cell conjugate formation can be visualized on FACS plots as cells positive for both cell tracker green and cell tracker orange dyes.

### In vitro cytotoxicity assay using enriched γδ T cells

Enriched γδ T cells (Effectors (E)) were co-cultured with CFSE-labelled Kasumi-3 cells (Targets (T)) at different E:T ratios in the presence of various concentrations of the bispecific antibodies for 24 h. At the end of the incubation period, 7-AAD (7-Aminoactinomycin D) (BioLegend, San Diego, CA) was added to the culture and cells were analyzed by a flow cytometer. Dead target cells were identified as 7-AAD^+^ FSC^low^ cells. To calculate bispecific antibody-mediated specific killing, cell lysis value from no bispecific antibody control was subtracted from the total cell death value obtained from the indicated bispecific antibodies. The spontaneous cytotoxicity of target cells was assessed by culturing them without effector cells or bispecific antibodies. EC_50_ was calculated using a 4-parameter dose-response curve with the concentration on the *x*-axis (log scale) and specific lysis on the *y*-axis (linear scale) using GraphPad Prism version 8.2.1 (La Jolla, CA). Log values on the *x*-axis were converted into anti-log values for representation purpose.

### In vitro activation, proliferation, and cytotoxicity assays using whole blood PBMCs

Briefly, CFSE-labelled PBMCs (0.1 − 0.2 × 10^6^ cells) were spiked in with 10,000 Kasumi-3 cells (at a ratio of 1:1 Vγ9^+^ γδ T cells and Kasumi-3 cells) and cultured in the absence or presence of indicated bispecific antibodies. Activation and proliferation of Vγ9^+^ γδ T cells were assessed by measuring the surface expression of CD69, CD25, and CFSE dilution, respectively, on day 3. For assessing bispecific mediated Vγ9^+^ γδ T cell cytotoxicity, PBMCs were spiked in with CFSE-labelled Kasumi-3 cells and cultured in the presence of the indicated bispecific antibodies. Elimination of Kasumi-3 cells (% 7-AAD^+^ cells among CFSE^+^ Kasumi-3 cells), as a measure of cytotoxicity, was measured on day 5 of the culture. Bispecific mediated Vγ9^+^ γδ T cells’ specific cytotoxicity was calculated by deducting the basal cytotoxicity, as described in the above sections. Procedures for AML patient-derived PBMCs are described in the Supplementary information.

### Cytokine and effector molecule analysis

For intracellular cytokine and effector molecules’ assessment, cells were initially surface stained with the indicated monoclonal antibodies, fixed, and permeabilized using BD Fix/Perm kit (BD Biosciences, San Jose, CA) as per the manufacturer’s instructions. Permeabilized cells were probed with monoclonal antibodies against intracellular cytokines (TNFα, IFNγ) or effector molecules (Granzyme B, Perforin) for 30 min at 4 °C. Cells were acquired on a flow cytometer. For assessing the cytokines, culture supernatants were collected at the indicated time points and subjected to quantification using a customized human magnetic Luminex assay 15 plex kit (R&D systems, Minneapolis, USA), as per the manufacturer’s instructions. Quantification of the cytokines was carried out in a MagPix multiplex detection system with xPONENT software (version 4.2).

### Xenograft tumor model and imaging of γδ T cells

The in vivo efficacy of the Vγ9/CD123 bispecific antibody was analyzed using a xenograft mouse model, as reported earlier with minor modifications [9, 16–19]. Detailed description of the experimental procedure for efficacy and imaging study is available in the Supplementary information.

## Results

### Vγ9^+^ γδ T cells are suitable as effector cells for redirection

We focused our studies on circulating γδ T cells that express heterodimers of Vγ9/Vδ2 TCR chains because they manifest potent anti-cancer functions [20]. The frequency of circulating Vγ9^+^ γδ T cells in the blood ranged from ~1 to 15% of total CD3^+^ T cells (Supplementary Fig. S1a), with an average of ~4% of Vγ9^+^ γδ T cells among the total T cells (Supplementary Fig. S1b). We further characterized the phenotype of Vγ9^+^ γδ T cells by stimulating them with Zoledronic acid that selectively expands and activates Vγ9^+^ γδ T cells from whole PBMCs. Majority of the Vγ9^+^ γδ T cells present in fresh PBMCs are either central memory (CD27+, CD45RA−) or effort memory (CD27−, CD45RA−) whereas activated cells develop into effector memory cells (CD27^−^, CD45RA^−^), express high levels of intracellular Granzyme B and Perforin, and upregulate expression of activation markers (Supplementary Fig. S1c–e). Collectively these data suggest that abundance of Vγ9^+^ γδ T cells in the circulation and phenotype of activated Vγ9^+^ γδ T cells is appropriate for redirecting these cells to kill tumor cells. We observed upregulation of some exhaustion markers on activated Vγ9^+^ γδ T cells (Supplementary Fig. S1f). However, these cells are perfectly functional (see the following sections) and show robust activity. Moreover, it is yet to be determined if the traditional markers of exhaustion apply to γδ T cells. It is still possible that these activated cells may become exhausted and we need to use caution in interpreting these data. Indeed, if exhaustion markers are expressed, then it opens the potential for synergy with anti-checkpoint inhibitors.

### Anti-TRGV9/anti-CD123 (Vγ9/CD123) bispecific antibody selectively binds to Vγ9^+^ γδ T cells and CD123-expressing tumor cells

**Fig. 1:**
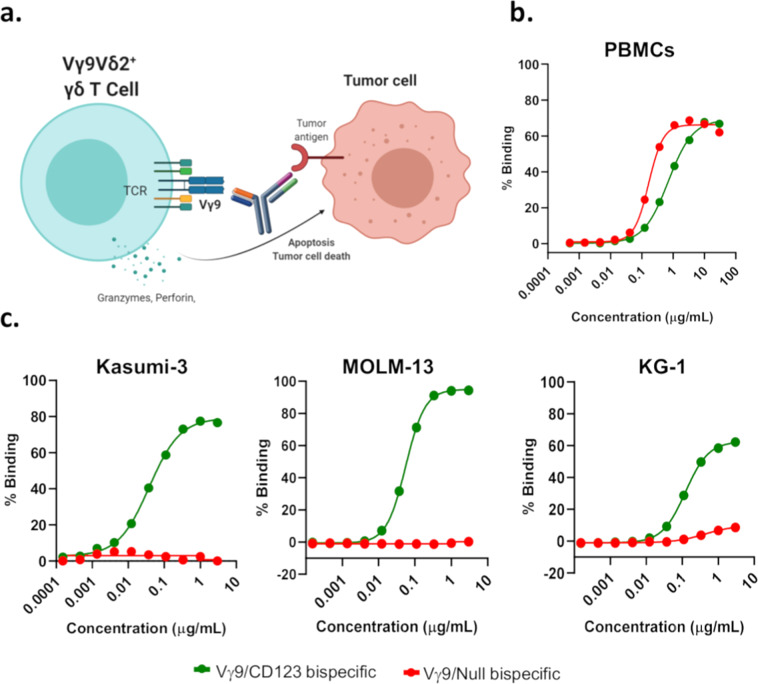
Mechanism of action and binding of anti-TRGV9/anti-CD123 (Vγ9/CD123) bispecific antibody to Vγ9^+^ γδ T cells and CD123-expressing tumor cells. **a** Bispecific antibody bridges the tumor to T cells and assists with the establishment of immune synapse, leading to the release of cytolytic agents such as perforin. Binding of bispecific antibodies to PBMCs (**b**) that were activated and expanded with Zoledronic acid+IL-2+IL-15 for 14 days and (**c**) CD123-expressing tumor cell lines (Kasumi-3, MOLM-13, and KG1) were assessed by flow cytometry. The mean EC_50_ value derived from seven healthy donors was 6.7 and 6.4 nM for Vγ9/CD123 (green) and Vγ9/Null (red) bispecific antibody, respectively, (**b**) and the mean EC_50_ value for tumor lines were 0.43, 0.51, and 0.94 nM for Kasumi-3, MOLM-13, and KG-1, respectively (**c**).

Vγ9/CD123 bispecific antibody was designed to recruit Vγ9^+^ T cells to kill tumor cells as shown in Fig. 1a (also see Supplementary Fig. S2). Binding of bispecific antibodies to Vγ9^+^ γδ T cells and CD123 expressing tumor cells (Kasumi-3, MOLM-13, and KG-1) was assessed; the data presented in Fig. 1b indicate binding of both Vγ9/CD123 and Vγ9/Null control bispecific antibodies to Vγ9^+^ γδ T cells. We further determined if Vγ9/CD123 bispecific antibody selectively binds to Vγ9^+^ γδ T cells by depleting Vγ9^+^ γδ T cells from enriched pan-T cells obtained from whole PBMCs and measuring the binding. Our data show the lack of binding of Vγ9/CD123 and null control bispecific antibodies to pan-T cells that were devoid of Vγ9^+^ cells (Supplementary Fig. S3a). Similarly, we determined binding of Vγ9/CD123 bispecific antibody to tumor cells; the data presented in Fig. 1c show binding of the Vγ9/CD123 bispecific antibody to CD123-expressing Kasumi-3, MOLM-13, and KG-1 tumor cells. Collectively, these data indicate that bispecific antibodies are suitable for recruiting Vγ9^+^ γδ T cells to eliminate CD123-expressing target cells.

### Vγ9/CD123 bispecific antibody recruits Vγ9^+^ γδ T cells into biphasic cell–cell conjugate formation with tumor cells

**Fig. 2:**
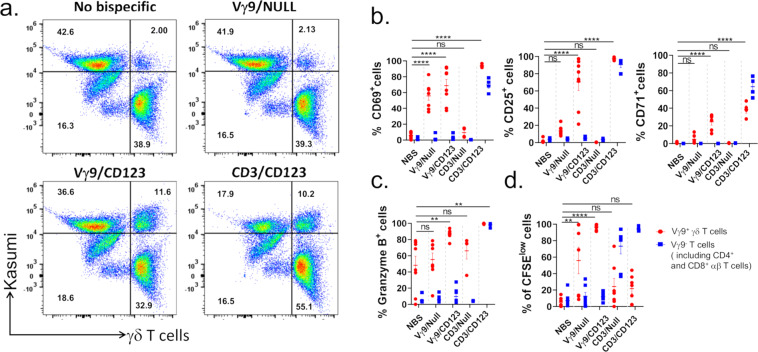
Vγ9/CD123 bispecific antibody mediates selective recruitment, activation differentiation, and proliferation of Vγ9^+^ γδ T cells. Cell trace green labelled enriched γδ T cells (effectors) were co-cultured with cell trace yellow labelled kasumi-3 cells (targets) at 1:1 ET ratio in the presence of 1 µg/mL of indicated bispecific antibody at 37 °C for 1 h. Cell–cell association was determined using flow cytometry and quantified as double positive cells in upper right quadrant of FACS plot (**a**). Numbers in quadrants indicate the frequency of respective population (**a**). **b** and **c** CFSE labelled Pan T-cells (effectors) from fresh PBMCs were co-cultured with Kasumi-3 cells (targets) at 1:1 ET ratio in the presence or absence of indicated bispecific antibodies at 37 °C for 72 h. Scatter plot graphs mirror the mean (±SEM) frequency of Vγ9^+^ γδ T cells and Vγ9^-^ γδ T cells (includes CD4^+^ and CD8^+^ T cells) that were positive for CD69 (left), CD25 (middle) and CD71 (right) surface expression (**b**), intracellular Granzyme B expression (**c**), and CFSE dilution (**d**). Red circle and blue square represent Vγ9^+^ γδ T cells and Vγ9^-^ T cells (includes CD4^+^ and CD8^+^ αβ T cells), respectively. Each dot represent data from an individual donor. The *p* values were calculated with a one-way ANOVA and Dunnett’s multiple comparison test (*p < 0.05, **p < 0.01, ***p < 0.001, ****p < 0.0001, and ns suggests *p* > 0.05). *NBS* No bispecific antibody.

In order to determine if Vγ9/CD123 bispecific antibody recruits Vγ9^+^ γδ T cells into biphasic cell–cell conjugate with target cells, γδ T cells (effector cells) and Kasumi-3 (Targets) cells were co-cultured in the presence of bispecific antibodies (Vγ9/CD123 and Vγ9/Null) and analyzed for conjugation formations. Data presented in Fig. 2a show that Vγ9/CD123 bispecific antibody mediated conjugation formation between Vγ9^+^ γδ T cells and CD123-expressing tumor cells, similar to the conjugation formation by anti-CD3/anti-CD123 (CD3/CD123) bispecific antibody. Taken together, these data suggest effective conjugation formation between effector and targets cells that is prerequisite for T cell-mediated cytotoxicity.

### Vγ9/CD123 bispecific antibody selectively activates Vγ9^+^ γδ T cells

To determine selective activation of Vγ9^+^ γδ T cells and induction of their cytotoxicity, we utilized a two cell co-culture system where enriched pan-T cells were co-cultured with Kasumi-3 cells at an ET ratio of 1:1 in the presence of bispecific antibody. To serve as positive and negative control, co-cultured cells were also stimulated with CD3/CD123 and Vγ9/Null bispecific antibodies. At the end of the culture period, the frequency of Vγ9^+^ and Vγ9^-^ γδ T cells (also includes CD4^+^ and CD8^+^ αβ T cells) positive for activation markers, CD69, CD25, and CD71 surface expression (Fig. 2b) and intracellular Granzyme B expression (Fig. 2c) and proliferation (Fig. 2d) was determined. Our data demonstrate that Vγ9/CD123 bispecific antibody selectively activates and mediates proliferation of only Vγ9^+^ γδ T cells, whereas CD3/CD123 bispecific antibody affects pan-T cells and mediates their activation.

### Vγ9/CD123 bispecific antibody efficiently mediates cytotoxicity induced by Vγ9^+^ γδ T cells

**Fig. 3:**
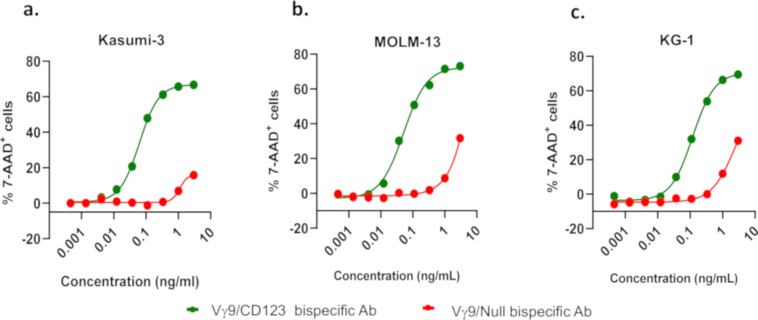
Vγ9/CD123 bispecific mediated γδ T cell cytotoxicity against CD123-expressing tumour cell lines. Healthy donor PBMCs were cultured with Zoledronic acid+IL-2+IL-15 for 14 days, for selective expansion of Vγ9^+^ γδ T cells. Day 14 PBMCs (effectors) were co-cultured with CFSE-labelled target (Kasumi-3/MOLM-13/KG-1 cells) cells at ET ratio 1:1 (by normalizing the ET ratio to Vγ9^+^ γδ T cell frequency in expanded PBMCs) in the presence of the indicated concentration of Vγ9/CD123 or Vγ9/Null arm control bispecific antibodies for a period of 16–24 h. Target cell lysis was determined by 7-AAD staining on flow cytometry. Mean EC_50_ value of Vγ9/CD123 (green) bispecific mediated lysis of Kasumi-3 (0.46 pM, panel **a**), MOLM-13 (0.66 pM, panel **b**), and KG1 (1.25 pM, panel **c**) cell lines. KG1 cell line was sensitive for bystander cytotoxicity by the Vγ9/Null (red) bispecific.

Vγ9^+^ γδ T cells (effectors) that were expanded from PBMCs in vitro were co-cultured with either Kasumi-3 cells or MOLM-13 or KG-1 cells (targets) in the presence of various concentrations of the bispecific antibodies, and cytotoxicity of the target cells was determined as described in “Materials and methods”. Our data show that Vγ9/CD123 bispecific antibody mediates Vγ9^+^ γδ T cell cytotoxicity against CD123-expressing kasumi-3, MOLM-13, and KG-1 cells in a dose-dependent manner (Fig. 3a–c). These data, thus, provide the proof of the concept that Vγ9/CD123 bispecific antibody can selectively activate and recruit Vγ9^+^ γδ T cells to effectively kill tumor cells.

### Vγ9/CD123 bispecific antibody potently mediates activation, proliferation, and cytotoxicity by stimulating Vγ9^+^ γδ T cell fraction among whole PBMCs

**Fig. 4:**
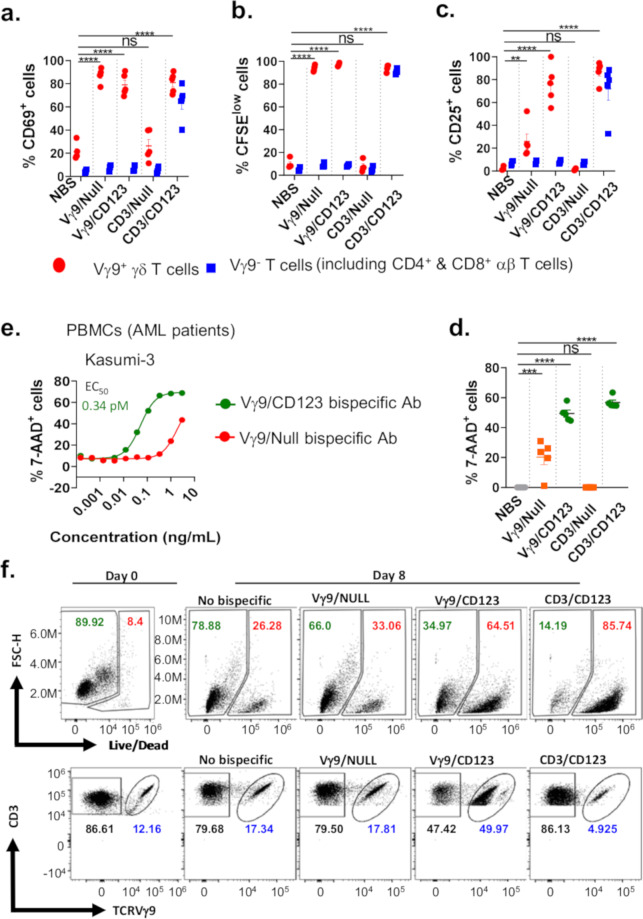
Vγ9/CD123 bispecific antibody mediates selective activation, proliferation, and effector functions of Vγ9^+^ γδ T cells among whole PBMCs. CFSE-labelled whole PBMCs from healthy individuals were cultured with Kasumi-3 cells in the presence or absence of the indicated bispecific antibodies at a concentration of 3 ng/mL. Scatter plot graphs mirror the mean (±SEM) frequency of Vγ9^+^ γδ T cells and Vγ9 TCR-depleted T cells that were positive for surface expression of CD69 (**a**), CFSE dilution (proliferation profile, **b**), CD25 (**c**), and the ability to eliminate exogenously spiked-in Kasumi-3 cells (cytotoxicity, **d**). The red circle and blue squares represent Vγ9^+^ (γδ) T cells and Vγ9 TCR-depleted T cells, respectively. Each dot represents data from an individual donor. (**e**) AML patient PBMCs cultured in the presence of Zol+IL-2+IL-15 for 14 days (effectors) were co-cultured with Kasumi-3 (target) cells at indicated concentrations of Vγ9/CD123 or Vγ9/Null arm control bispecific antibodies for 16–24 h. Target cell lysis was assessed by 7-AAD^+^ staining on flow cytometry. Cytotoxicity values represented in (**e**) are after subtracting cell death from no bispecific (NBS) antibody controls. Mean EC_50_ value of Vγ9/CD123 (green) bispecific mediated lysis of Kasumi-3 (0.34 pM) was from two patients. The control Vγ9/Null (red) bispecific antibodies showed non-target specific cytotoxicity (**e**). **f** Whole PBMCs from AML patients were cultured either in the presence or absence of indicated bispecific antibodies for 8 days. The number in the representative FACS dot plots refers to live and dead cells among AML blasts (**f**, upper row) and the frequency of Vγ9^−^ CD3^+^ and Vγ9^+ ^CD3^+^ cells among total CD3^+^ cells (**f**, lower row) on day 0 and 8 of the culture period. Representative data are means of values derived from two AML patients. The *p* values were calculated with a one-way ANOVA and Dunnett’s multiple comparison test. (*p < 0.05, **p < 0.01, ***p < 0.001, ****p < 0.0001, and ns suggests *p* > 0.05). *NBS* no bispecific antibody.

To assess whether Vγ9/CD123 bispecific is capable of selective stimulation of Vγ9^+^ γδ T cell fraction present in the whole PBMCs, we co-cultured whole PBMCs with CD123-expressing kasumi-3 cells in the presence or absence of Vγ9/CD123 and CD3/CD123 along with control bispecific antibodies. The frequency of Vγ9^+^ or Vγ9^-^ γδ T cells (Pan T cells lacking Vγ9^+^ γδ T cells) expressing activation markers CD69 and CD25 were determined by FACS analysis. Data presented in Fig. 4a, c show induction of surface expression of CD69 and CD25 by Vγ9/CD123 bispecific antibody in the Vγ9^+^ γδ T cell compartment, whereas CD3/CD123 activated both Vγ9^+^ and Vγ9^-^ T cell populations. Similarly, CFSE dilution, a measure of proliferation, was also very selective for Vγ9/CD123 bispecific antibody; it only induced proliferation of Vγ9^+^ γδ T cells (Fig. 4b). Conversely, the CD3/CD123 bispecific antibodies induce proliferation of T cells irrespective of Vγ9^+^ cells (Fig. 4b). We further determined the ability of Vγ9/CD123 bispecific antibody to eliminate exogenously added Kasumi-3 target cells among whole PBMCs. Our data suggest that although Vγ9/CD123 bispecific antibody only recruited and activated Vγ9^+^ γδ T cells (a fraction of total T cells in the PBMC population), it mediated efficient target cell elimination, similar to CD3/CD123, which recruits and activates all pan-T cells (Fig. 4d). To strengthen the concept further, Vγ9^+^ γδ T cell-depleted pan-T cells or total pan-T cells obtained from whole PBMCs were incubated in the presence and absence of bispecific antibodies at various concentrations and Kasumi-3 tumor cells (Supplementary Fig. S3). Selective binding of Vγ9/CD123 or Vγ9/Null bispecific antibodies is only shown when Vγ9^+^ cells were present (Supplementary Fig. S3a). Cytotoxicity mediated by Vγ9/CD123 bispecific antibody was determined and is presented in Supplementary Fig. S3b, where no cytotoxicity was seen when Vγ9^+^ γδ T cells were depleted (Supplementary Fig. S3b, left panel). On the other hand, the cytotoxicity of CD3/CD123 bispecific antibody with pan-T cells and pan-T cells depleted of Vγ9^+^ γδ T cells was evident (Supplementary Fig. S3b, right panel).

### Vγ9/CD123 bispecific antibody efficiently induces cytotoxicity by stimulating Vγ9^+^ γδ T cell fraction among AML patient PBMCs

Three approaches were adopted to assess the ability of anti-TRGV9 targeting bispecific antibodies in redirecting Vγ9^+^ γδ T cells obtained from AML patients to kill tumor cells. In the first approach in vitro expended Vγ9^+^ γδ T cells obtained from an AML patient were studied for cytotoxicity against Kasumi-3 cells, where Vγ9/CD123 bispecific antibody mediated efficient cytotoxicity against CD123-expressing Kasumi-3 cells (Fig. 4e). These data, thus, provide the proof of the concept that Vγ9/CD123 bispecific antibody can selectively activate and recruit Vγ9^+^ γδ T cells from AML patients to kill tumor cells.

In the second approach, we assess the ability of Vγ9^+^ γδ T cells present in the AML patient PBMCs for elimination of AML blasts present within the PBMCs by culturing AML patient’s whole PBMCs in the presence of Vγ9/CD123 for 8 days and counting the number of dead cells at the end of the culture. Since AML blasts are CD123-positive, they can potentially be targeted by Vγ9/CD123. The data depicted in Fig. 4f show that Vγ9/CD123 bispecific antibody, but not its null control, effectively mediated the elimination of endogenous blasts (represented by the increased fraction of dead cell compartments among the CD3 negative fractions) (upper row). Further, Vγ9/CD123 bispecific antibody selectively induced the proliferation of Vγ9^+^ γδ T cells as measured by the frequency of Vγ9^+^ γδ T cells among total T cells (Fig. 4f, lower row). The frequency of elimination of endogenous blasts seems to be synchronized with the proliferation of Vγ9^+^ γδ T by Vγ9/CD123 bispecific. These data suggest that Vγ9/CD123 bispecific antibody can efficiently activate and recruit Vγ9^+^ γδ T cells from AML patients to kill endogenous AML blasts.

In the third approach, we spiked the AML patient PBMCs with DLL3-expressing SHP-77 cells in the presence or absence of Vγ9/DLL3 bispecific antibody. Data depicted in Supplementary Fig. S4a show that Vγ9/DLL3 bispecific antibody effectively mediated the elimination of exogenously added target cells. Further, Vγ9/DLL3 bispecific antibody mediates elimination of target cells even at low ET ratios, i.e., when one effector cell (Vγ9^+^ T cells) is dominated by 5–10 target cells. Similarly, Vγ9/DLL3 bispecific antibody induced the proliferation of Vγ9^+^ γδ T cells (Supplementary Fig. S4b), as evident by the increase in the absolute numbers of recovered Vγ9^+^ γδ T cells. Taken together, our data suggest efficient and selective activation and induction of cytotoxicity mediated by Vγ9/CD123 bispecific antibody despite the presence of a low number of Vγ9^+^ γδ T cells among total T cells in PBMC population in AML patients.

### Vγ9^+^ γδ T cell selective redirection does not elicit cytokine storm compared to pan-T cell re-direction

**Fig. 5:**
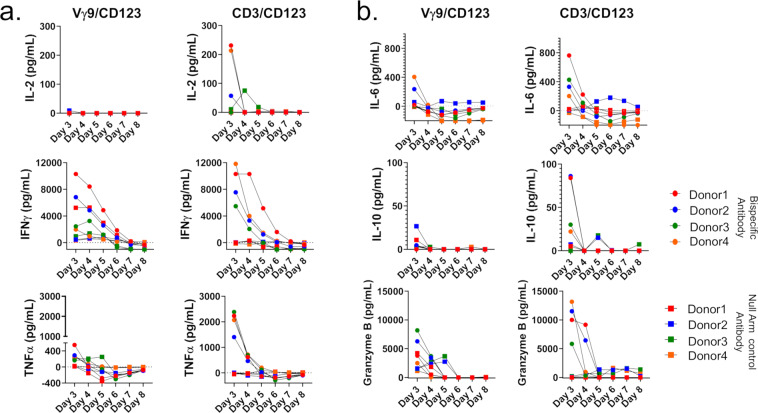
Vγ9^+^ γδ T cell selective redirection does not elicit cytokine storm compared to Pan-T cell re-direction. Whole PBMCs from healthy individuals were cultured with kasumi-3 cells in the presence or absence of the indicated bispecific antibodies (3 ng/ml) as described in Fig. 4. From day 3 of culture onward, 100 µL of culture medium was removed every day from the wells, without disturbing the cells, and replenished with fresh medium until day 8 of culture. Cytokines were assessed from day 3 to day 8 cell culture supernatant. **a**, **b**, Concentration of various cytokines or effector molecules in the culture supernatant of whole PBMCs stimulated with the indicated bispecific antibodies. Red, blue, green, and orange circles and squares represent PBMCs from four individual donors stimulated with indicated bispecific antibodies or NULL arm control bispecific antibodies, respectively. Representative data of *n* = 4 donors from one independent experiment are shown here.

We compared Vγ9/CD123 bispecific antibody with CD3/CD123 antibody-induced cytokine production by whole PBMCs in the presence of Kasumi-3 cells and bispecific antibodies as described in “Materials and methods”. From day 3 to day 8 of culture supernatants were analyzed for cytokine production. Data presented in Fig. 5a, b indicates that Vγ9/CD123 bispecific antibody induced much lower cytokines as compared to CD3/CD123 bispecific antibody, notably, IL-6 and IL-10 that are believed to the main players for cytokine storm in patients undergoing CD3-redirection immunotherapy. Based on these data, Vγ9-redirection therapy is less likely to induce cytokine storm, and thus, it may help to broaden the therapeutic index.

### Vγ9/CD123 bispecific antibody effectively controlled KG-1 tumor cell growth in a xenograft model

**Fig. 6:**
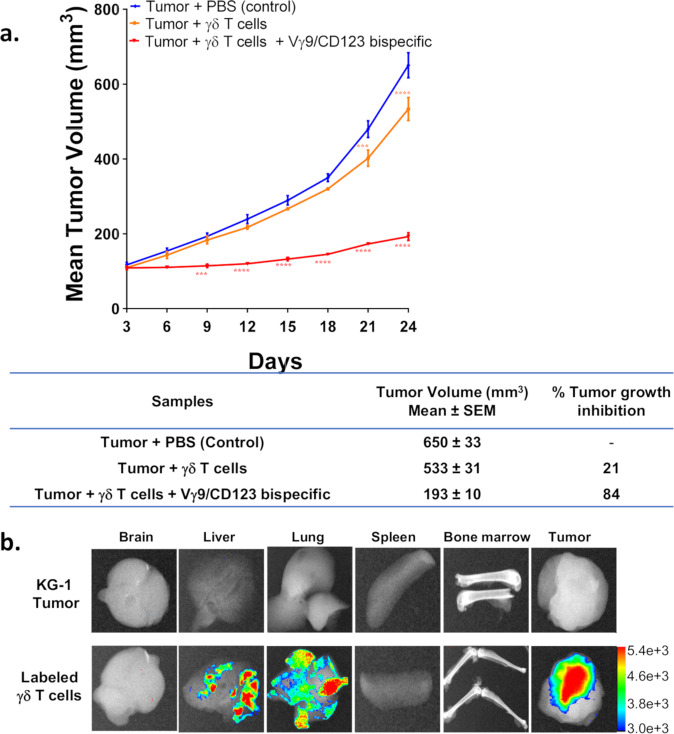
Innate homing and Vγ9/CD123 bispecific mediated anti-tumor activity of Vγ9^+^ (γδ) T cells in KG-1 xenograft model. **a** NOD-SCID mice were engrafted subcutaneously with KG-1 cells and were treated with PBS alone or expanded γδ T cells alone or expanded γδ T cells Vγ9/CD123 bispecific antibody. Expanded γδ were infused to the mice very week for four times. The table represents the percentage of tumor growth inhibition for all groups on day 24 of the experiment. **b** Fluorescence intensity in representative images refers to the abundance of adoptively transferred Dil dye-labelled Vγ9^+^ γδ T cells in various organs of mice with established KG-1 tumors (~1000 mm^3^). *n* = 6 mice for each group from a single experiment and 2–4 mice from two independent experiments. Statistical significance was calculated using two-way ANOVA followed by Bonferroni’s post-test. *p* ≤ 0.05 were considered as a statistically significant difference between groups (*p < 0.05, **p < 0.01, ***p < 0.001, ****p < 0.0001, and ns suggests *p* > 0.05).

A xenograft NOD SCID mouse model with subcutaneously injected KG-1 tumor cell line and γδ T cells was used to evaluate the efficacy of Vγ9/CD123 bispecific antibody. Our data presented in Fig. 6a indicate that selective recruitment of Vγ9^+^ γδ T cells by Vγ9/CD123 bispecific antibody induced significant tumor growth inhibition and efficacy as compared to control treatment. In parallel, to understand the innate homing capacity of activated Vγ9^+^ γδ T cells to tumors, Dil dye-labelled γδ T cells were adoptively transferred into mice with established KG-1 tumors (~1000 mm^3^), and 24 h post injection, harvested organs confirmed predominant homing of Vγ9^+^ (γδ) T cells into the tumor, albeit a small fraction of these cells was also found in other organs in some mice (Fig. 6b). To further validate the utility of Vγ9/CD123 we performed pharmacokinetic (PK) study in C57BL/6 mice. PK analysis revealed *t*_1/2_ values of 151 ± 56 h (6.29 ± 2.3 day) and 148 ± 88 h (6.16 ± 3.6 day) for Vγ9/CD123 and Vγ9/Null bispecific antibodies, respectively, at a dose of 1 mg/kg and 120 ± 12 h (5 ± 0.5 day) and 120 ± 31 h (5 ± 1.2 day), respectively, at a dose of 10 mg/kg, indicating a half-life suitable for redirection (Supplementary Fig. S5).

Collectively, our data provide the in vivo proof of the concept that γδ T cells can efferently and rapidly home to tumors, and Vγ9/CD123 bispecific antibody can selectively activate and recruit Vγ9^+^ γδ T cells to effectively kill tumor cells, and Vγ9/CD123 bispecific antibody could serve as a viable redirection approach for AML patients.

## Discussion

Although CD3 T cell redirection-based immunotherapies have become effective treatments for B cell-related malignancies, they still remain to be validated for AML patients. Nonetheless, several T cell engagers (TCEs) are being tested in the clinic for AML [6, 21]. Despite the promise of CD3-based TCEs, they manifest high toxicity and lack efficacy in many cases. This could be attributed to an intrinsic feature of these engagers that recruit and activate all kinds of T cells irrespective of the phenotype or lineage. For example, CD19 TCE, blinatumomab, activates T-regulatory cells that limit the activity of CD8 cytotoxic T cells to proliferate and effectively kill tumor cells [5], and increases T regulatory cell numbers that correlate with non-responsiveness to blinatumomab in ALL patients. Similarly, solitomab, an EpCAM TCE, showed serious adverse events with dose-limiting toxicities across all dose levels tested in Phase I clinical trials that prohibited dose escalation to the required therapeutic level [22]. Data from large number of trials with TCEs suggest that cytokine response syndrome is a key challenge for TCE therapies [23]. Activation of pan T-cells by anti-CD3 is likely the cause of massive of cytokine release seen in the patients.

To address some of these issues we employed a strategy to recruit and activate a specific Vγ9Vδ2^+^ circulating γδ T-cell subset to target tumor cells. Our data suggest that Vγ9/CD123 bispecific antibody stimulated these cells appropriately to activate and induce cytotoxic response to AML blasts. Our in vivo imaging studies revealed that infused Vγ9^+^ γδ T cells selectively colonized to tumors, which provides additional advantage for exerting efficacy in the presence of Vγ9/CD123 bispecific antibody, further favoring this subset. In addition, Vγ9Vδ2 T cells express DNAM-1 and are known to kill autologous AML blasts via the perforin/granzyme pathway by recognizing the ligands for DNAM-1 that are expressed by AML cells [24]. This function of Vγ9Vδ2 T cells may help to control tumor escape even when they lose target antigens.

Although selective recruitment of a subset of γδ T cells is appealing, it is not clear whether engagement of a small population of γδ T cells would form sufficient contacts with tumor cells or induce effective anti-tumor response. The data presented here clearly demonstrate that Vγ9/CD123 bispecific antibody can efficiently recruit Vγ9^+^ γδ T cells to form cell–cell conjugates of γδ T cells with tumor cells, selectively activates only Vγ9^+^ γδ T cell and mediates cytotoxicity against tumor cells at low E:T ratios in vitro suggesting potent killing ability and that small numbers are sufficient to effectively eliminate tumor cells. As only a small population of T cells are activated, we anticipate this will result in overall lower cytokine release as compared to polyclonal T cell activation using CD3-redirection approaches. This concept is also validated in vivo where anti-TRGV9/anti-CD123 bispecific antibody mediates γδ T cell cytotoxicity against KG1 tumors and controls their growth.

One of the key questions is whether autologous Vγ9^+^ γδ T cells will be enough to eliminate the overwhelming population of AML blasts, and whether this subset of γδ T cells from AML patients are fully functional. In this report, we first demonstrated that Vγ9/CD123 bispecific antibody selectively induced the proliferation of Vγ9^+^ γδ T cells from AML patients, and second, we showed that Vγ9/CD123 bispecific antibody efficiently activated and recruited Vγ9^+^ γδ T cells to effectively kill endogenous AML blasts and AML tumor cell lines. In addition, anti-TRGV9/anti-DLL3 bispecific antibody was effective at low ET ratios (1:8), suggesting bispecific antibody-mediated serial killing activity by γδ T cell from AML patients. Thus, we believe, despite the small number of Vγ9^+^ γδ T cells compared to AML blasts, there are multiple ways that these cells could mount a productive immune response against overwhelmed blasts in AML patients.

Although the Vγ9^+^ subset of γδ T cells could also serve as a good candidate for γδ CAR-T cells, there are distinct advantages for redirecting these cells with Vγ9/CD123 bispecific antibody. First, γδ T cells are innate immune cells, γδ CAR-T cells may not persist for very long in vivo as has been demonstrated recently, where γδ CAR-T cell infusion resulted in limited persistence of these cells in animal models [25]. This may require multiple infusions and developing a large supply of the allogenic product of γδ CAR-T cells. On the other hand, Vγ9/CD123 bispecific antibody can be easily dosed as frequently as needed to achieve optimal efficacy. Second, there is a clear safety advantage; in the case of toxicity induced by the Vγ9/CD123 bispecific antibody, it can easily be mitigated by simply withdrawing or adjusting the dose of the drug as opposed to γδ CAR-T cells where the only option would be to manage the patient with complicated procedures or develop a product that can easily be eliminated from the system. Third, as outlined elegantly by Cummins et al., the general limitation of CAR-T cells in AML is that the targeting myeloid antigens may lead to prolonged myeloablation, which is not clinically tolerable [26]. Fourth, CAR-T cells may directly contribute to target cell antigen loss, resulting in low antigen density. In a recent study, it was demonstrated that CAR-T cells decreased antigen density on target cells through the mechanism of trogocytosis, a process by which targeted antigens are transferred to the CAR-T cells [27]. This process not only decreased antigen density on the target cells, but also makes CAR-T cells positive for the tumor antigen; once the CAR-T cells obtain the target antigen, they themselves can become targets of CAR-T fratricide, potentially contributing to lack of persistence. Finally, the prime advantage for the bispecific antibody approach is the ease and cost of treatment for AML patients. Although some of the limitations of γδ CAR-T cells could potentially be mitigated by further development of the CAR-T field, it would be difficult to predict if there is a clear winner until clinical data comparing these two approaches become available.

In summary, we present a mechanism to recruit and activate the Vγ9^+^subset of γδ T cells to mediate cytotoxicity against AML blasts. Selective recruitment of γδ T cells, particularly activation of only a small subset of γδ T cells, should only lead to overall low cytokine release. As recruited cells will be highly potent and readily penetrate tumor tissues, this combined with the low overall cytokine production should lead to a broader therapeutic index. Furthermore, such a γδ T cell targeted therapy would present a major advantage for most AML patients.

## Supplementary information

Supplementary
