# Supplementary material for: Selective recruitment of γδ T cells by a bispecific antibody for the treatment of acute myeloid leukemia
Source: Leukemia. 2021 Feb 1;35(8):2274–84. doi: 10.1038/s41375-021-01122-7 (PMC8324575; doi:10.1038/s41375-021-01122-7)
Supplement: Supplementary file 1 — Supplementary [file 41375_2021_1122_MOESM1_ESM.pdf]

Supplementary

Selective recruitment of γδ T cells by a bispecific antibody for the treatment of Acute Myeloid Leukemia

[1]Rajkumar Ganesan, [2]Vijaykumar Chennupati, [2]Balaji Ramachandran, [1]Michael Riis Hansen, [1]Sanjaya Singh, [1*]Iqbal S. Grewal

Supplementary Materials and Methods

Human Blood sample collection:

Blood sample were obtained from both healthy individuals and Acute Myeloid Leukemia (AML) cancer patients following informed consent. Approval for blood collection from human subjects and subsequent usage for bio medical research was obtained from Ethical committees of participating clinical centers. Diagnosis of AML was performed based on French-American-British (FAB) classification system. Written informed consent was obtained from all the human subjects for the use of the data generated by their samples.

Cell lines and reagents:

Cell lines (Kasumi-3, MOLM-13 and KG1; acute myeloblastic leukemia cell lines and SHP-77; prostate epithelial cell line) used in this study were purchased from ATCC. Kasumi-3 cells were cultured in RPMI-1640 medium supplemented with 20% FBS and 1x Pen/Strep. MOLM-13 and SHP-77 cells were cultured in RPMI-1640+10%FBS +1x Pen/Strep medium. KG-1 cells were cultured in Iscove's Modified Dulbecco's Medium (IMDM) supplemented with 20% FBS and 1x Pen/Strep. All cell culture media and supplements were purchased from Gibco (Thermo Fisher Scientific Inc, Waltham, MA). CFSE (Carboxyfluorescein succinimidyl ester) and 7-AAD (7-Aminoactinomycin D) reagents were obtained from ThermoFisher scientific and BioLegend respectively. Recombinant antigen (Vγ9-Vδ2-Fc) protein was prepared as the extracellular domain of heterodimer human Vγ9-Vδ2 TCR and containing a C-terminal human IgG1 Fc tag that was expressed as a secreted protein in ExpiCHO cell line. Purification protocol was identical to the one routinely used for mAbs, except that the final protein was dialyzed into PBS pH 6.8. Purity was determined by SDS-PAGE and SEC to 99.5%.

Engineering of the anti-TRGV9-based bispecific antibody

The mouse IgG1 anti-human T cell receptor TRGV9 clone 7A5 was obtained from Abcam (cat # ab171109). The amino acid sequence of 7A5 was determined by LC-MS/MS analysis (performed by Lake Pharma, San Carlos, CA). 7A5 was humanized for engineering targeting constructs following the approach outlined by Singh et al [1]. Based on sequence conservation, the heavy chain germline IGHV1-8*01 was chosen for framework adaption (Supplementary Fig. S1a). Three somatic hypermutation sites in the heavy chain were chosen for binary library design. A potential Iso-Asp isomerization site (DG motif) in the CDR-H2 was also included in the design to mitigate this potential liability. For light chain frame adaption, IGKV4-1*01 was chosen as the closest homologous human germline. Owing to high sequence homology, only one position (Asn22) was included in the library design. The variants were cloned and expressed in *E. coli*. The supernatants were screen in single point ELISA and the periplasmic preparation was used for dose response. A mouse/human chimeric 7A5 Fab was used as parental control and named 7A5_17. Clone 7A5_17 maintained the binding activity similar to murine 7A5 and was converted to IgG for additional profiling.

Binding kinetics for 7A5 to TCR Vγ9-Vδ2-Fc was determined by SPR. Data was obtained using ProteOn XPR36 Surface Plasmon Resonance (SPR) System from BioRad. Goat anti-murine Fc surface was immobilized on a GLC chip, and binding was tested by capturing the 7A5-17 at different densities. The monovalent Vγ9-Vδ2 heterodimer fused to human Fc construct flew in to bind in solution at 0.3 µM in 3-fold dilution series. Association and dissociation times was set to 4 min and 30 min, respectively. Raw binding data were processed by double referencing: 1) interspot on an empty chip surface; 2) column 6 where no 7A5 was captured, to monitor the noise to background of the antigen binding to the GAM-Fc capture surface. Data was global fitted to a

1:1 simple Langmuir binding model. The binding affinity for clone 7A5_17 was determined to be 1.9 nM (Supplementary Fig. S1b).

The variable region sequence of 7A5_17 (anti-TRGV9) and I3RB217 (anti-CD123 antibody) or an anti-DLL3 antibody were used to generate bispecific antibodies. Anti-TRGV9/anti-DLL3 (Vγ9/DLL3), anti-TRGV9/anti-CD123(Vγ9/CD123) and anti-TRGV9/Null arm (Vγ9/Null) bispecific antibodies were produced as full-length antibodies in the knob-into-hole format as human IgG1 or IgG4, as previously described [2]. Nucleic acid sequences encoding variable regions were sub-cloned into a custom mammalian expression vectors containing constant region of IgG1or IgG4 expression cassettes using standard PCR restriction enzyme based standard cloning techniques. The bispecific antibodies were expressed by transient transfection in Chinese Hamster Ovary cell line. The antibodies were initially purified by Mab Select SuRe Protein A column (GE Healthcare) [3]. The column was equilibrated with PBS pH 7.2 and loaded with fermentation supernatant at a flow rate of 2 mL/min. After loading, the column was washed with 4 column volumes of PBS followed by elution in 30 mM sodium acetate, pH 3.5. Fractions containing protein peaks as monitored by absorbance at 280 nm were pooled and neutralized to pH 5.0 by adding 1% 3 M sodium acetate pH 9.0. The bispecific mAbs were further purified on a preparative Superdex 200 10/300 GL (GE healthcare) size exclusion chromatography (SEC) column equilibrated with PBS buffer. The integrity of sample was assessed by endotoxin measurement and SDS-PAGE under reducing and non-reducing conditions.

HDX epitope mapping of 7A5 mAb on TCR Vγ9 protein

The procedures used to analyze the mAb perturbation were carried out as previously described [4-6] with minor modifications. Recombinant human Vγ9-Vδ2 was incubated with and without anti-

Vγ9 7A5 mAb (in BSA-free PBS pH 7.2 buffer) in 118 µL deuterium oxide labeling buffer (50 mM sodium phosphate, 100 mM sodium chloride at pD 7.4) at 10 °C. At time points 0 sec, 10 sec, 60 sec, 300 sec, 1800 sec or 7200 sec, hydrogen-deuterium exchange (HDX) mixture was quenched by adding 130 µL of 4 M guanidine hydrochloride, 0.85 M TCEP buffer followed by a 3 min incubation at 10 °C. Final pH is ~2.5. The quenched samples were subjected to online pepsin/protease XIII digestion using an in-house packed pepsin/protease XIII column (2.1 x 30 mm). The resultant peptides were analyzed using an UPLC-MS system comprised of a Waters Acquity UPLC coupled to a Q Exactive™ Hybrid Quadrupole-Orbitrap Mass Spectrometer (Thermo). The peptides were separated on a 50 x 1 mm C8 column with a 16.5 min gradient from 2-31% solvent B (0.2% formic acid in acetonitrile). Solvent A was 0.2% formic acid in water. Peptide identification was done through searching MS/MS data against the Vγ9-Vδ2 TCR sequence with Mascot. The mass tolerance for the precursor and product ions were 7 ppm and 0.02 Da, respectively. Raw MS data was processed using HDX WorkBench, software for the analysis of HDX MS data [7]. The deuterium levels were calculated using the average mass difference between the deuterated peptide and its native form (t0). Extent of protection was inferred by measuring the differences in hydrogen/deuterium exchange between Vγ9-Vδ2 TCR alone or in complex with the 7A5. The protection map and further refinements of the analysis showed significant protection of regions identified as residues: $L_{49}VSISYDGTVRKESGIPSGK_{68}$ on human Vγ9-Vδ2 TCR upon complexation with 7A5 (Supplementary Fig. S1c). The epitope mapping by HXMS indicated that the 7A5 antibody primarily bound to a portion of CDR2 and FR3 in the Vγ9 chain of the TCR. We also determined the paratope of this antibody/antigen complex. Nearly all CDRs of 7A5 in making contribution to the Vγ9-Vδ2 TCR binding (Supplementary Fig. S1d).

PBMC Isolation:

Peripheral Blood Mononuclear Cells (PBMCs) were isolated from healthy donors. Briefly, whole blood was diluted in plain RPMI-1640 medium at 1:1 ratio and carefully layered onto Lymphoprep^{TM} gradient (STEMCELL Technologies, Vancouver, Canada) in a 50 mL falcon Tube (Corning, NY, USA). Centrifuged the tube at 450xg for 30 min at room temperature with acceleration and deacceleration was kept at 0. After centrifugation, cells were collected from the interface and erythrocyte lysis was performed using RBC lysis buffer (Sigma, St. Louis, MO) for 5 min at room temperature. Supernatant containing lysed erythrocyte was discarded and the cell pellet was washed twice with plain RPMI-1640 medium. After washes, cells were resuspended in culture medium (RPMI-1640+10%FBS+1x Pen/Strep), counted and used it for downstream applications or frozen down in the freezing medium (90%FBS+ 10%DMSO) at a density of $25x10^6$ cells/mL and stored in liquid nitrogen until further use.

Isolation and expansion of Vγ9$^+$ $\gamma\delta$ T cells from whole PBMCs

Selective expansion of Vγ9$^+$ $\gamma\delta$ T cells from whole PBMCs was achieved using Zoledronic acid. PBMCs were isolated from healthy individual or cancer patient's blood, as described in earlier section. For selective expansion of Vγ9$^+$ $\gamma\delta$ T cells, isolated PBMCs were cultured in culture medium (RPMI-1640 containing 10%FBS, 1x Pen/Strep) supplemented with recombinant human IL-2 (rhIL-2) (to a final conc of 1000, 400 and 100 IU/mL on day 0, 2 and rest of the 14 day culture period respectively), recombinant human IL-15 (rhIL-15) to a final concentration of 10ng/mL(on all days of the culture period) and Zoledronic acid to a final conc of 5μM (on day 0) for 14 days. Culture medium containing IL-2 and Il-15 was replenished once in 2-3 days and the cultures were transferred into new flasks as necessitated by the growth of cells. $\gamma\delta$ T cells were negatively enriched from day 14 PBMCs stimulated with Zol+IL-2+IL-15 (or from day 0 PBMCs) using

EasySep™ Human γδ T cell isolation kit, as per the manufacturer's instructions. Enriched γδ T cells were verified for their purity by staining the cells, before and after enrichment, with monoclonal antibodies against TCRγδ, TCRVγ9 and TCRαβ.

Flow cytometry:

All flow cytometry studies were carried out on Novocyte flow cytometer (ACEA biosciences, Singapore) and data was analyzed by FlowJo analysis software (Treestar Inc, Ashland, OR). All antibodies used in this study were purchased from BioLegend (San Diego, CA), unless and until specified. Supplementary Table 1 refers to the detailed list of antibodies used in this study. For surface staining, 0.1-0.2 million cells were initially Fc blocked with human TruStain FcX™ (Biolegend,) in the 100 µL culture medium (RPMI-1640+10%FBS+1xPen/Str) for 20 minutes at 4°C. Washed twice with wash buffer (PBS+2%FBS) and stained with LIVE/DEAD™ Fixable violet dye (Thermo Fischer Scientific Inc, Waltham, CA) in 100 µL PBS for 20 minutes at room temperature. Alternatively, cells were incubated in PBS containing human TruStain FcX™ and LIVE/DEAD™ Fixable violet dye at 4°C for 30 minutes. For surface staining, cells were stained in 100 µL of wash buffer (PBS+2%FBS) with antibodies specific for cell surface antigens for 30 minutes at 4°C. After incubation period, cells were washed twice with wash buffer and acquired on Novocyte flow cytometer immediately. Alternatively, cells were fixed with BD Cytofix (BD Biosciences) for 30 minutes at 4°C, washed twice with wash buffer, resuspended in wash buffer (PBS+2%FBS) and acquired on flow cytometer within 24-48 h of fixation. For intracellular staining, surface stained cells were fixed and permeabilized by resuspending them in 100µL BD Cytofix/Cytoperm (BD Biosciences) and incubated the cell suspension at 4°C for 15-30 min in dark. After two washes with 200µL of 1x Perm/Wash buffer, cells were probed by incubating them in 100µL of Perm/Wash buffer containing antibodies against intracellular effector molecules

(Granzyme B and Perforin) at 4°C for 30 min in dark. After incubation period, cells were washed twice with 1x perm wash and resuspended in wash buffer (PBS+2%FBS) and acquired on Flow cytometer. All flow cytometry-based experiments reported in the present study were performed in a 96-well V- bottom plates. Majority of the flow cytometry experiments were performed in a blinded manner, where the test compound and/or sample identity was masked and/or replaced by numbers.

Bispecific antibodies Pharmacokinetics in C57BL/6 mice:

Female C57BL/6 mice (6-7 weeks old) were procured from Envigo and were randomized into groups based on the body weight. Mice were then dosed intraperitonially with Vγ9/CD123 and Vγ9/Null bispecific antibodies at a concentration of 1 mg/kg or 10 mg/kg per body weight. Blood was drawn from the retro-orbital puncture of the dosed mice at hours 1, 6, 24, 72, 120, 168 and 288 post dosing. Serum was collected from the derived blood and stored at -80°C until further use. As a control, blood was drawn from mice injected with vehicle (PBS) control alone and was used as a control. Total Vγ9/CD123 and Vγ9/Null arm bispecific antibodies concentration in mouse serum was quantified using a Sandwich-based ELISA. For quantifying Vγ9/CD123 bispecific antibody, purified human CD123 extracellular domain (ECD) was passively adsorbed to a Nunc MaxiSorp™ flat bottom microtiter plate (ThermoFisher Scientific) for overnight at 4°C. After washes and non-specific binding block, diluted mouse serum sample was added to purified human CD123 ECD protein adsorbed plates for two hours at room temperature. After washes, plate captured bispecific antibody was detected by incubating the plate with horseradish (HRP)-conjugated Goat Anti-Human IgG (Fcγ fragment specific) antibody (Jackson ImmunoResearch laboratories, USA) for 2 hours at room temperature. For quantifying anti-TRGV9/Null bispecific antibody in mouse serum, purified Vγ9Vδ2-Fc fusion protein was adsorbed to Nunc MaxiSorp™

flat bottom plates for overnight at 4°C. After washes and non-specific binding blocking, mouse serum sample was allowed to bind to purified Vγ9-Vδ2-Fc fusion protein coated plates for two hours at room temperature. Bound bispecific anti-TRGV9/Null bispecific antibody was detected using biotinylated Respiratory Syncytial Virus-Fusion protein (RSV-F), followed by its detection using Streptavidin-HRP (R&D systems). HRP activity was quantified by the addition of chromogenic HRP-substrate,3,3', 5,5'-tetramethylbenzidine (TMB) (Sigma) and the resulting activity was read at OD450 and OD 570 nM on Spectramax Multimode plate reader. Standard controls were prepared by spiking in the various concentration of bispecific antibodies (anti-TRGV9/anti-CD123 and anti-TRGV9/Null arm) in PBS containing mouse serum to mirror the diluted mouse serum samples. All samples, including standards and tests, were quantified in duplicates. The concentration of the bispecific antibodies in the serum samples was extrapolated from a four-variable fit of the standard curve using GraphPad Prism (version 8.2.1). The maximum serum concentration (Cmax), serum elimination half-life ($t_{1/2}$) and Area under the curve (AUC) were estimated using Phoenix™ WinNonlin® (Certara, CA, USA) software (version 8.2).

***In vitro* activation, proliferation and cytotoxicity assays using whole blood PBMCs:**

<u>*For AML patient's PBMCs:*</u>

AML patient's PBMCs ($0.3x10^6$) were cultured with GM-CSF (2.5ng/mL), IL-3 (6.25ng/mL), SCF (3ng/mL) and FLT3L (2.5ng/mL) in the presence or absence of indicated bispecific antibodies. Recombinant IL-15 (10ng/mL) was added to the culture 24 h after the initiation and cultured for a period of 8 days. Bispecific antibody mediated $V\gamma9^+$ γδ T cells cytotoxicity of blast cells was measured by assessing the frequency of dead cells among $CD3^-$ cells in the absence or presence of indicated bispecific antibodies on day 8. Abundance of $V\gamma9^+$ γδ T cells was also measured as the frequency among total $CD3^+$ cells in the absence or presence of indicated

bispecific antibodies on days 0 and 8. In an alternative approach, AML patient's PBMCs (~0.25 x 10^6 cells) were cultured with CFSE labelled SHP-77 target cells expressing Delta-like ligand 3 (DLL3), at an ET ratio of 1:1 (normalized to Vγ9$^+$ γδ T cells in PBMCs) for a period of 6 days in the culture medium containing IL-2 (100 IU/mL), IL-15(10 ng/mL) and IL-18(100 ng/mL) in the presence or absence of anti-TRGV9/anti-DLL3 (Vγ9/DLL3) bispecific antibody. Cytotoxicity of target cells was assessed by measuring the frequency of dead SHP-77 cells (% 7-AAD$^+$ cells among CFSE$^+$ labelled targets) on day 6 of the culture. Proliferation of Vγ9$^+$ γδ T cells were measured by assessing the frequency and absolute number among total T cells. Absolute numbers of Vγ9$^+$ γδ T cells were calculated by adding in the CountBright™ Absolute counting beads (Invitrogen, Carlsbad, CA) in the staining panel.

Xenograft Tumor model and Imaging of γδ T cells

Experiments were performed in strict accordance with rules approved by an in-house animal committee (IAEC SYNGENE/IAEC/1140/02-2020). Five to six weeks old NOD/MrkBomTac-Prkdc^{scid} (NOD SCID-F) mice (Vivo Biotech Ltd. Hyderabad, India) received a single s.c. injection of 5x10^6 KG-1 cells, mixed with 1:1 ratio of Matrigel, in the right flank. Two days post s.c. injection, mice were randomized based on body weight and segregated into three groups of 6 mice each. After the randomization, where indicated, mice received expanded Vγ9$^+$ γδ T cells injection subcutaneously on day 2 (2.5 x 10^6/mouse), day 7 (8 x 10^6/mouse), day 14 (4.5 x 10^6/mouse), and day 23 (6 x 10^6/mouse). Subcutaneous injection of either 15 µg/kg IL-2 in PBS or with 1.5 mg/kg bispecific antibody (Vγ9/CD123) was carried out that were repeated weekly for a total of 4 weeks. Body weight and tumor volume were measured every three days. Pharmacokinetics (PK) of bispecific antibodies was determined in C57BL/6 mice as described above. Ten million expanded Vγ9$^+$ γδ T cells (~95% Vγ9$^+$ cells) labelled with 0.5 µM DiI dye

(Molecular Probes, Eugene, OR) were injected intravenously into NOD-SCID mice bearing established KG-1 tumors (~1000 mm^3). 24 hrs. after cell transfer all animals were euthanized and organs (Brain, lung, liver, spleen, bone marrow, tumor) were collected for imaging. Fluorescence Modality was carried out to measure the signal intensity as a method of detecting transferred γδ T cells in various organs using imaging machine (Burker, Billerica, MA)

Statistical analysis

Statistical significance among multiple groups was calculated using one-way ANOVA and Dunnett's Test. For tumor growth inhibition, two-way ANOVA followed by Bonferroni's post-test was performed using Graph Pad Prism (Version 8.3.0). p values ≤ 0.05 were considered as a statistically significant difference between groups. * indicates $p<0.05$, ** $p<0.01$, *** $p<0.001$, **** $p<0.0001$ and ns suggests $p>0.05$.

Supplementary Figure Legend

Supplementary Figure S1: Comprehensive characterization of Vγ9^{+} γδ T cells.

Representative FACS plots show the frequency of Vγ9^{+} γδ T cells (TCRVγ9^{+}CD3^{+}) cells among the whole PBMCs. Numbers in quadrants represent the frequency of respective population (**a**). Scatter dot plot graph summarizes the frequency (mean±SEM) of Vγ9^{+} γδ T cells among whole PBMCs of healthy individuals (data from six donors) (**b**). Each dot represent data from a healthy individual (**b**). Scatter plot graph summarizes the frequency of cells positive for Naïve (CD27^{+}CD45RA^{+}), Central memory (CD27^{+} CD45RA^{-}), Effector Memory (CD27^{-} CD45RA^{-}) and Effector Memory cells that re-expresses CD45RA (EMRA, CD27^{-}CD45RA^{+}) phenotypes of resting (day 0) and activated (day 14) Vγ9^{+} γδ T cells (**c**). Each dot represent data from individual donors (**c**). (**d-f**) Numbers in representative FACS plots refer to the mean (±SEM) frequency of Vγ9^{+} γδ T cells on day 0 (top row) and day 14 (bottom row) of PBMC culture that are positive for GzmB and Perforin (**d**), CD62L, CD69, CD44, CD71 and CD45RO (**e**) PD1, CTLA4, 2B4, Lag3, Lag3, TIGIT and Tim3 surface expression (**f**). n=12 and 7 donors from resting Vγ9^{+} γδ T cells (day 0) and n= 14 and 7 donors from activated Vγ9^{+} γδ T cells (day 14) for Granzyme B (GzmB) and perforin intracellular expression respectively. n= 7 donors for CD62L, CD69, CD44, 2 donors for CD45RO and CD71 surface expression on day 0 Vγ9^{+} γδ T cells. n= 8 donors for CD62L, 9 donors for CD69 and CD44, 5 donors for CD45RO and CD71 surface expression on activated Vγ9^{+} γδ T cells (day14). n= 13 donors for PD1 and Lag3, 5 donors for CTLA4 and 2B4, 4 and 7 donors for TIGIT and Tim3 surface expression respectively on resting Vγ9^{+} γδ T cells (day 0). n=16 donors for PD1, 5 donors for CTLA4 and 2B4, 13 donors for Lag3, 14 donors for TIGIT surface expression on activated Vγ9^{+} γδ T cells (day 14).

>5 individual experiments were carried out. Statistical significance (*p* values) was calculated with one-way ANOVA and Dunnett's multiple comparison test for **c** (* indicates $p<0.05$, ** $p<0.01$, *** $p<0.001$, **** $p<0.0001$ and ns suggests $p>0.05$).

Supplementary Figure S2. Humanization and Characterization of mouse anti-human TCR Vγ9 clone (7A5). (**a**). Based on sequence homology, we chose germline IGHV1-8*01 and IGKV4-1*01 for framework adaption. Humanization of murine clone 7A5 was performed following the process outlined by Singh et al[24]. A potential Iso-Asp isomerization site (DG motif) was also included in the design (**b**). Binding kinetics of mouse anti-human TCR Vγ9 [clone 7A5] and recombinant Vγ9-Vδ2-Fc antigen by SPR at 25 °C. Experimental data (black line) and 1:1 Langmuir binding fitting (red line) is shown. The association phase between (first ~250 sec) is follow by the dissociation phase. Global fitting to a 1:1 simple Langmuir binding model resulted $k_{on} = 1.3 \pm 0.2 \times 10^{5}$ M^{-1} S^{-1} and $k_{off} = 2.43 \pm 0.3 \times 10^{-4}$ S^{-1} giving a $K_D = 1.9$ nM. (**c**). HX-MS epitope mapping for the mouse anti-human TCR Vγ9 [clone 7A5] mAb and Vγ9/Vδ2 fused to human Fc. Peptide region comprising of L_{49}VSISYDGTVRKESGIPSGK_{68}, was protected by mAb 7A5. A molecular model (using crystal structure PBD: 1HXM) of TCR Vγ9-Vδ2 and residues in the epitope are highlighted in the sphere representation (**d**). HDX paratope mapping on the murine clone 7A5. A molecular model of the Fab with residues in the paratope are highlighted.

Supplementary Figure S3. Anti-TRGV9/anti-CD123 bispecific antibody selectively binds and mediates γδ T cell cytotoxicity.

a. Green, blue and Red, magenta lines reflect the binding of Vγ9/CD123 and Vγ9/NULL bispecific antibodies respectively at indicated concentrations to pan-T cells (green and red lines) and pan-T cells depleted of $V\gamma9^{+}$ γδ T cells (Magenta and blue lines). **b.** Graphs show the frequency of target cell lysis (% $7\text{-}AAD^{+}$ cells) mediated by Vγ9/CD123 or CD3/CD123 bispecific and respective NULL arm control antibodies at indicated concentrations, upon co-culture of pan-T cells (green and red lines) and pan-T cells depleted of $V\gamma9^{+}$ γδ T cells (Magenta and blue lines) with target (Kasumi-3) cells.

Supplementary Figure S4. Anti-TRGV9 bispecific antibody efficiently redirects γδ T cells of AML patient PBMCs. AML patient PBMCs were cultured with CFSE labelled SHP-77 cells (Targets) and were cultured in the absence or presence of anti-TRGV9/anti-DLL3 (Vγ9/DLL3) bispecific antibody in culture medium containing IL-2+IL-15+IL-18 cytokines for 6 days. (**a**) Bars mirror the frequency (±SEM) of $7\text{-}AAD^{+}$ target cells (specific target cell killing) at various Effector (**E**) and Target (**T**) ratios that were cultured in the absence or presence of Vγ9/DLL3 bispecific antibody. (**b**) Bars refer to the absolute number of $CD3^{+}V\gamma9^{+}$ cells recovered on day 6 from the cultures shown in (**a**). Representative data was derived from two AML patients from two independent experiments for a and one donor for b.

Supplementary Figure S5. Serum half-life of anti-TRGV9/anti-CD123 (Vγ9/CD123) and anti-TRGV9/Null (Vγ9/Null) bispecific antibodies.

Green and Red lines refer to the mean (±SD) concentrations of Vγ9/CD123 and Vγ9/Null bispecific antibodies respectively in serum at various time points after injecting C57BL/6 mice with 1 mg/kg (**a**) or 10 mg/kg (**b**) body weight concentration of indicated bispecific antibodies.

n=3 mice for 1,6 and 24 h time point and 6 animals for 72, 120, 168 and 288 h time point, respectively. (**c**) values listed in table refer to half-life ($t_{1/2}$), area under clearance (AUC) and maximum concentration (C_{max}) of indicated bispecific antibodies at a dose of 1 mg/kg or 10 mg/kg.

Supplementary Fig. S1

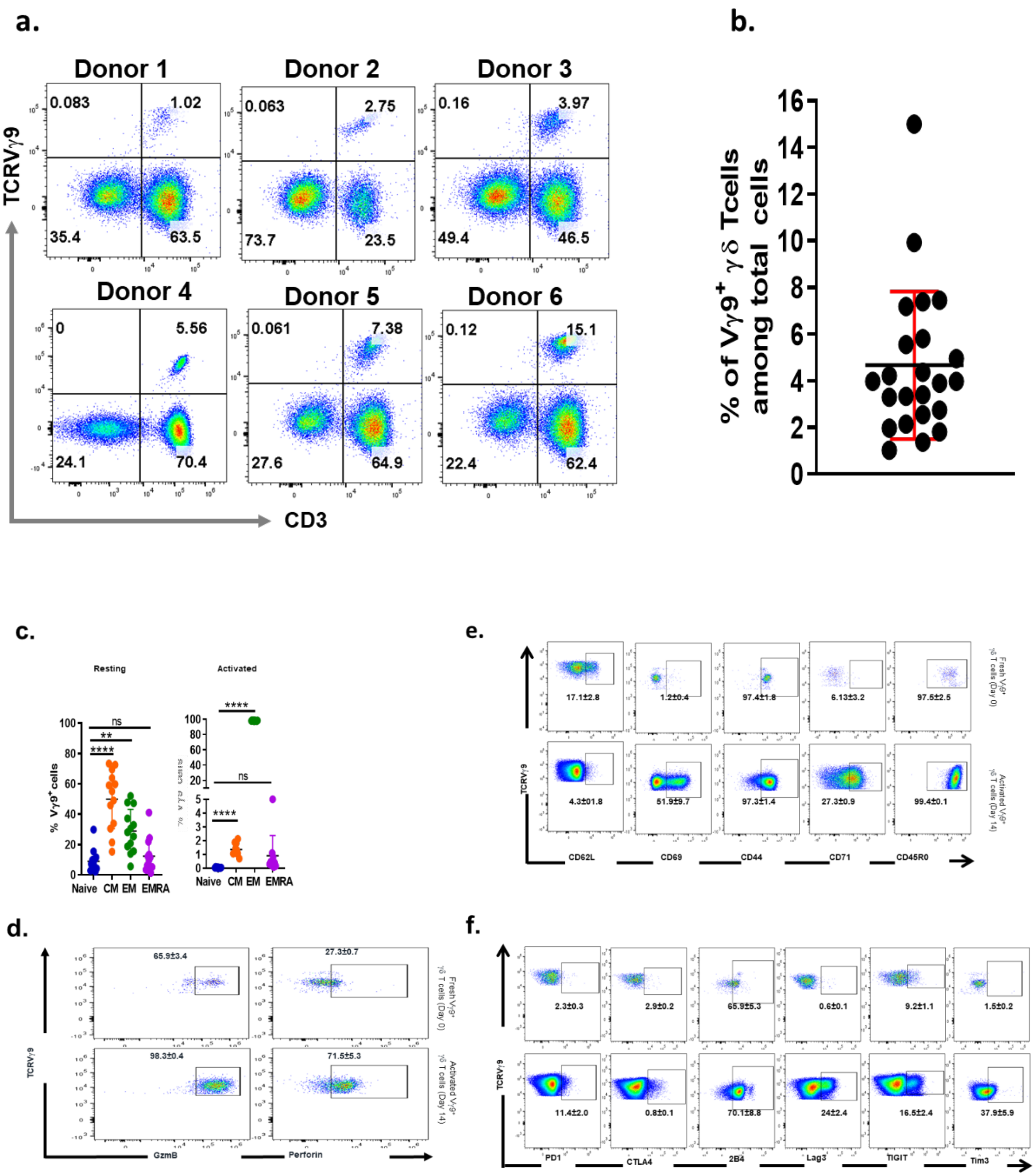

Supplementary Fig. S2

a

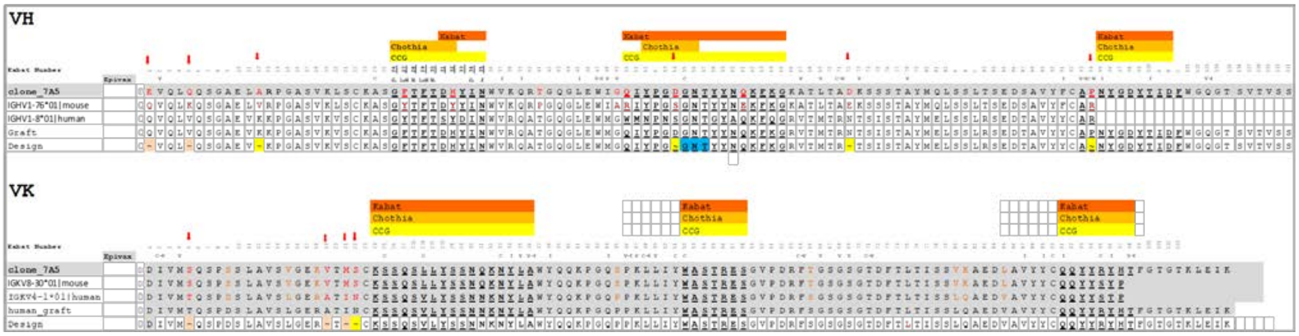

b

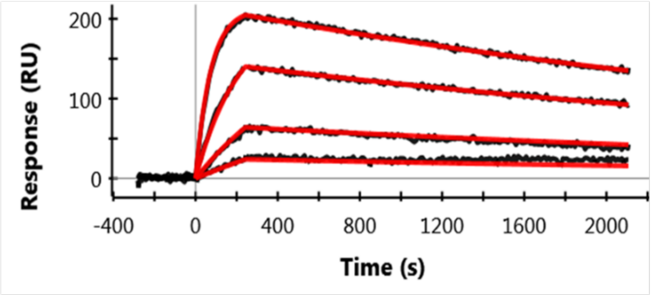

Table -1

k_{on} ($M^{-1} s^{-1}$)	k_{off} (s^{-1})	K_D (nM)
$1.3 \pm 0.2 \times 10^5$	$2.43 \pm 0.3 \times 10^{-4}$	1.9 ± 0.5

Supplementary Fig. S3

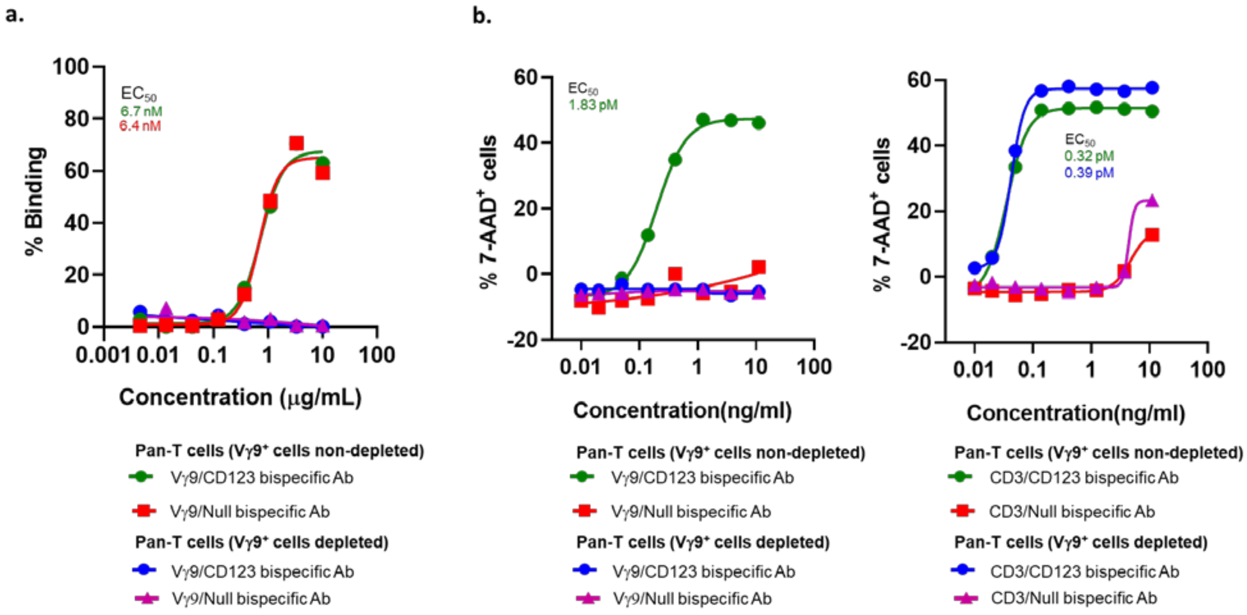

Supplementary Fig. S4

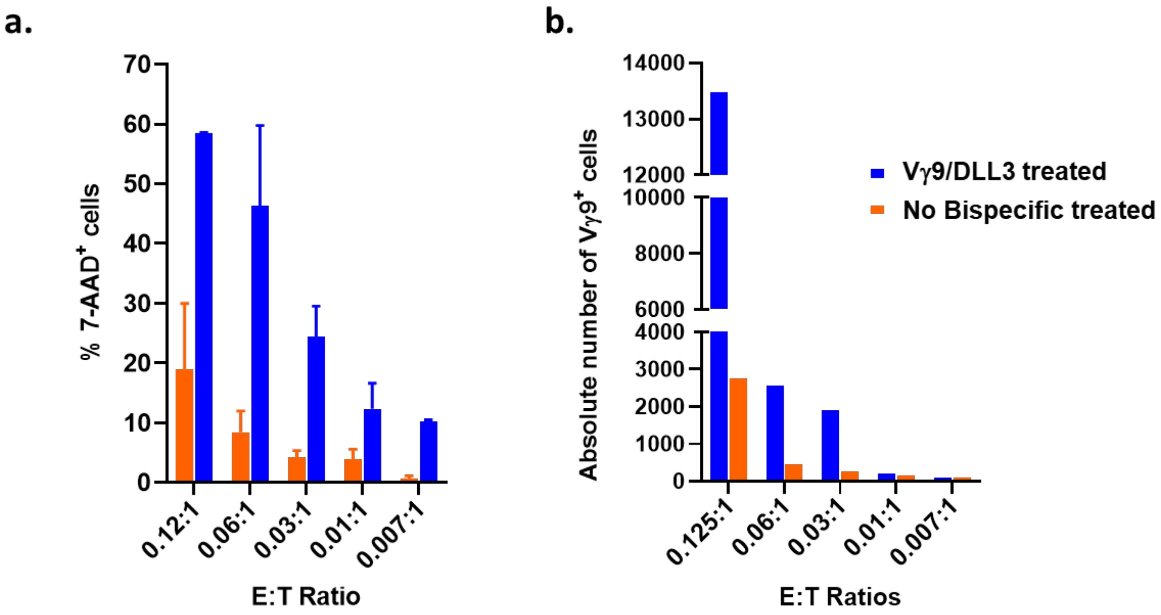

Supplementary Fig. S5

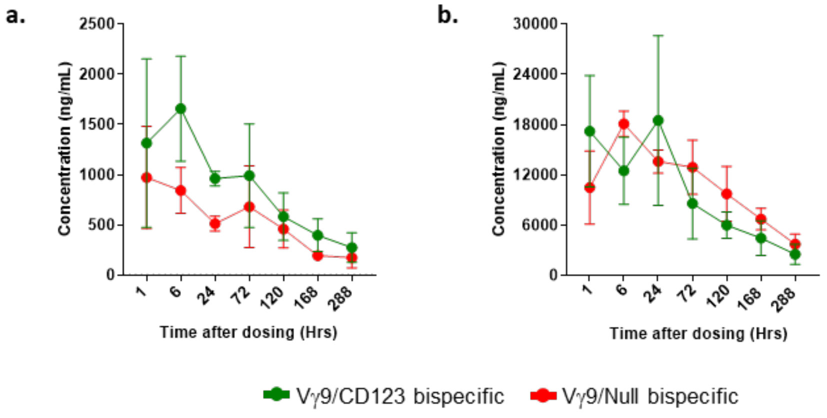

c.

S.No	bispecific Ab	Administered dose (mg/Kg)	$t_{1/2}$ (h)	AUC (h*ng/mL)	C_{max} (ng/mL)
1	Vγ9/CD123	1	151±56	153000±65600	1230±575
2	Vγ9/Null	1	148±88	98800±30900	1010±447
3	Vγ9/CD123	10	120±12	1630000±874000	15000±9370
4	Vγ9/Null	10	120±31	2270000±284000	16800±1730

h: hour, max: Maximum, C: concentration

Reference

1. Singh S, Kroe-Barrett RR, Canada KA, Zhu X, Sepulveda E, Wu H, *et al.* Selective targeting of the IL23 pathway: Generation and characterization of a novel high-affinity humanized anti-IL23A antibody. *mAbs* 2015; **7**(4)**:** 778-791.

2. Atwell S, Ridgway JB, Wells JA, Carter P. Stable heterodimers from remodeling the domain interface of a homodimer using a phage display library. *J Mol Biol* 1997 Jul 4; **270**(1)**:** 26-35.

3. Brown NL, Bottomley SP, Gore MG. Affinity purification of human IgG using immobilised, mutated immunoglobulin-binding domains from protein A of Staphylococcus aureus. *Biochem Soc Trans* 1998 Aug; **26**(3)**:** S249.

4. Huang RY, Krystek SR, Jr., Felix N, Graziano RF, Srinivasan M, Pashine A, *et al.* Hydrogen/deuterium exchange mass spectrometry and computational modeling reveal a discontinuous epitope of an antibody/TL1A Interaction. *mAbs* 2018 Jan; **10**(1)**:** 95-103.

5. Horn JR, Kraybill B, Petro EJ, Coales SJ, Morrow JA, Hamuro Y, *et al.* The role of protein dynamics in increasing binding affinity for an engineered protein-protein interaction established by H/D exchange mass spectrometry. *Biochemistry* 2006 Jul 18; **45**(28)**:** 8488-8498.

6. Hamuro Y, Coales SJ, Southern MR, Nemeth-Cawley JF, Stranz DD, Griffin PR. Rapid analysis of protein structure and dynamics by hydrogen/deuterium exchange mass spectrometry. *J Biomol Tech* 2003 Sep; **14**(3)**:** 171-182.

7. Pascal BD, Willis S, Lauer JL, Landgraf RR, West GM, Marciano D, *et al.* HDX workbench: software for the analysis of H/D exchange MS data. *J Am Soc Mass Spectrom* 2012 Sep; **23**(9)**:** 1512-1521.
